# Role of carglumic acid in the long-term management of propionic and methylmalonic acidurias

**DOI:** 10.1186/s13023-024-03468-4

**Published:** 2024-12-18

**Authors:** Sufin Yap, Serena Gasperini, Shirou Matsumoto, François Feillet

**Affiliations:** 1 Department of Inherited Metabolic Diseases, Sheffield Children’s Hospital, Sheffield Children’s NHS Foundation Trust, Western Bank, Sheffield, S10 2TH UK; 2 Metabolic Rare Disease Unit “Fondazione Mariani”, Pediatric Department, Fondazione IRCCS San Gerardo dei Tintori, Monza, Italy; 3 Department of Neonatology, Kumamoto University, Honjo 1-1-1, Chu-oh-ku, Kumamoto, Japan; 4 Pediatric Unit, Reference Center for Inborn Errors of Metabolism, University Hospital of Nancy, INSERM UMR_S 1256, Nutrition, Genetics, and Environmental Risk Exposure (NGERE), Faculty of Medicine of Nancy, University of Lorraine, Nancy, France

**Keywords:** Carglumic acid, Expert opinion, Long-term management, Methylmalonic aciduria, Propionic aciduria

## Abstract

Propionic aciduria (PA) and methylmalonic aciduria (MMA) are rare inherited disorders caused by defects in the propionate metabolic pathway. PA due to propionyl coenzyme A carboxylase deficiency results in accumulation of propionic acid, while in MMA, deficiency in methylmalonyl coenzyme A mutase leads to accumulation of methylmalonic acid. Hyperammonemia is related to a secondary deficiency of N-acetylglutamate (NAG), the activator of carbamoyl phosphate synthetase 1, which is an irreversible rate-limiting enzyme in the urea cycle. Carglumic acid (CGA) is a synthetic structural analog of human NAG and is approved for the treatment of patients with hyperammonemia due to PA or MMA. CGA is well tolerated and its use in normalizing ammonia levels during acute hyperammonemic episodes in patients with PA and MMA is well established. This expert opinion analyzed clinical evidence for CGA and discussed its place, along with other management strategies, in the long-term management of PA or MMA. A literature search of PubMed was undertaken to identify publications related to the chronic use of CGA, transplantation, dietary management, ammonia scavengers, and gene therapy for treatment of patients with PA or MMA. The authors selected the most relevant studies for inclusion. Four clinical studies, one single center case series, and three case reports show that CGA is safe and effective in the chronic treatment of PA and MMA. In particular, the addition of CGA is associated with a reduction in hyperammonemic decompensation episodes and admission to hospital, compared with conventional dietary treatment alone. Current treatment guidelines and recommendations include the use of CGA mainly in acute decompensation, however, lag in considering the benefits of long-term CGA treatment on clinical and biochemical outcomes in patients with PA or MMA. CGA is safe and effective in the chronic treatment of PA and MMA and may help to resolve some of the issues associated with other strategies used to treat these disorders. Thus, CGA appears to have potential for the chronic management of patients with PA and MMA and should be recommended for inclusion in the chronic treatment of these disorders.

## Introduction

Propionic aciduria (PA) and methylmalonic aciduria (MMA) are rare inherited disorders arising from defects in the propionate metabolic pathway. Deficiencies in propionyl coenzyme A carboxylase and methylmalonyl coenzyme A mutase enzymes, respectively, result in accumulation of propionic and methylmalonic acids [1, 2]. The buildup of these organic acids leads to toxicity in the brain and other organs [3]. The overall incidences of PA and MMA in Western populations have been estimated at up to 1/150,000 and ~ 1/50,000 births, respectively, although the incidences are much higher in some countries [4].

**Fig. 1:**
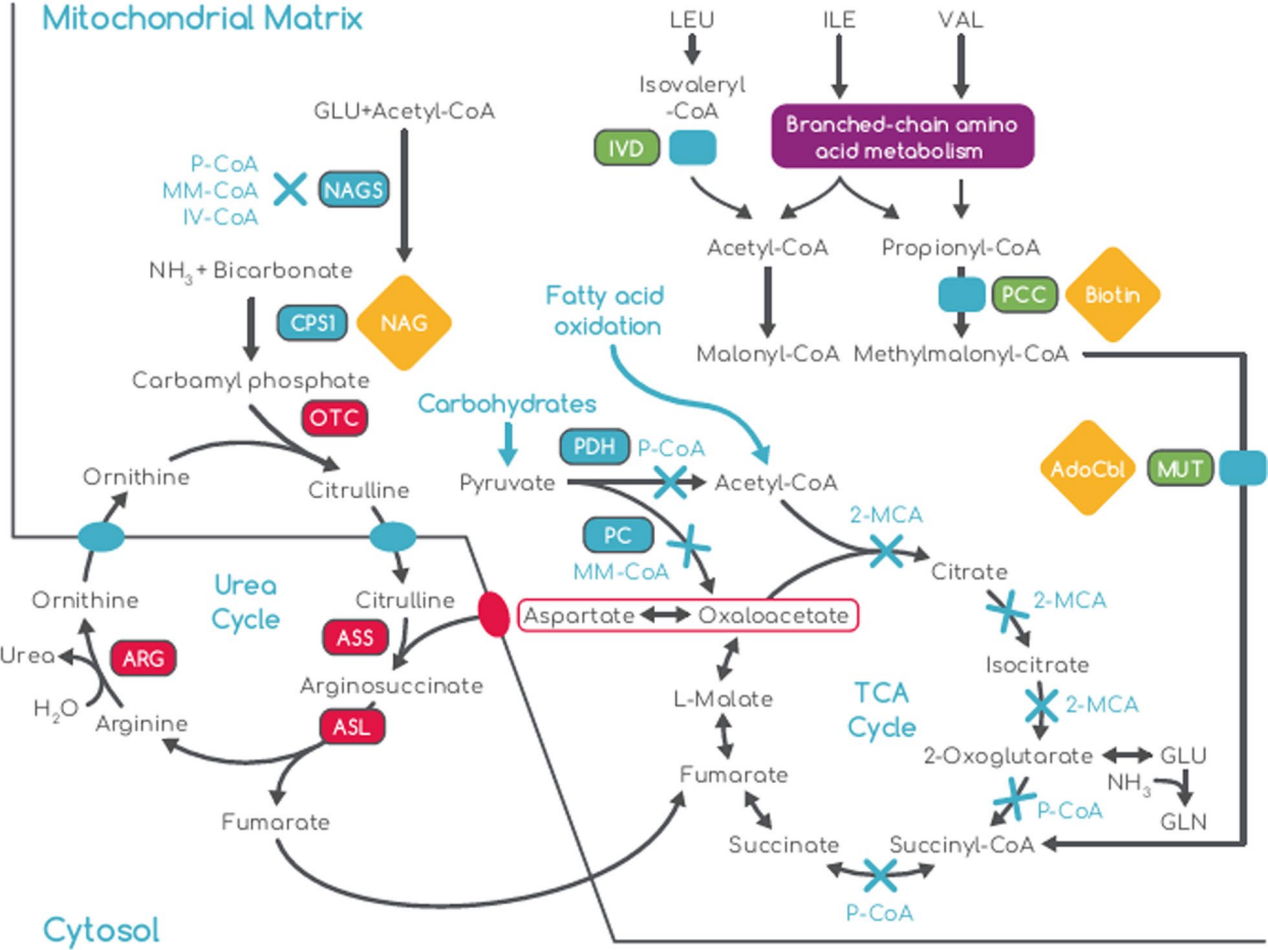
The biochemical pathways involved in pathogenesis of propionic aciduria and methylmalonic aciduria. Adapted with permission from Haberle et al., 2008 [59] (Creative Commons Attribution 4.0 International License (http://creativecommons.org/licenses/by/4.0/). Rectangles indicate key affected enzymes: red rectangles indicate the primary enzymes affected in urea cycle disorders (ornithine transcarbamylase, argininosuccinate synthetase, argininosuccinate lyase, and arginase); green rectangles indicate the primary affected enzymes in OAs (propionyl-CoA carboxylase, methylmalonyl-CoA mutase, isovaleryl-CoA dehydrogenase); blue solid rectangles are positions of primary enzyme blocks. Blue crosses indicate secondary enzyme inhibition; blue texts are enzyme precursors; orange diamonds are key enzyme co-factors. 2-MCA, 2-methylcitrate; CoA, coenzyme A; ARG, arginase; ASL, argininosuccinate lyase; ASS, argininosuccinate synthetase; CPS1, carbamoyl phosphate synthetase-1; GLN, glutamine; GLU, glutamate; H2O, water; IV-CoA, isovaleryl-CoA; IVD, isovaleryl-CoA dehydrogenase; LEU, leucine; MM-CoA, methylmalonyl-CoA; MUT, methylmalonyl-CoA mutase; NAG, N-acetylglutamate; NAGS, N-acetylglutamate synthase; NH3, ammonia; OTC, ornithine transcarbamylase; PC, pyruvate carboxylase; PCC, propionyl-CoA carboxylase; P-CoA, propionyl-CoA; PDH, pyruvate dehydrogenase complex; TCA, tricarboxylic acid; VAL valine

Propionyl coenzyme A carboxylase and methylmalonyl coenzyme A mutase enzymes catalyze different steps in the propionate pathway, from which breakdown products of some amino acids (valine, isoleucine, and to a lesser extent threonine and methionine), odd-chain fatty acids, and cholesterol, are recruited into the Krebs cycle [3]. A deficiency in these enzymes causes accumulation of their metabolites, leading to coenzyme A depletion and secondary cataplerosis in the Krebs cycle, which is associated with low levels of glutamate and glutamine in PA and MMA [3]. This in turn inhibits synthesis of N-acetylglutamate (NAG). NAG is an activator of carbamoyl phosphate synthetase 1 (CPS1), an irreversible rate-limiting enzyme of the urea cycle; thus, reduced NAG synthesis leads to secondary hyperammonemia (Fig. 1) [1, 5, 6].

Because of the accumulation of toxic metabolites, energy deprivation, and hyperammonemia, patients with PA and MMA usually have poor outcomes [3]. PA and MMA are complicated by acute life-threatening metabolic crises [4, 7]. These crises are precipitated by energy deprivation and can lead to organ failure and death if left untreated [7]. Acute symptoms of PA and MMA include neonatal metabolic encephalopathy, recurrent ketoacidosis, coma or Reye-like syndromes, psychomotor retardation, seizures, and feeding difficulties [4, 7]. Symptoms can also occur in the absence of acute crises [7]. Chronic presentation includes neurological complications, failure to thrive, cardiomyopathy (mainly in PA), and renal failure (almost exclusively in MMA) [4].

Hyperammonemia in PA and MMA (manifesting as hypotonia, seizures, emesis, headache, psychosis, and abnormal neurological changes) can be life threatening and result in brain damage that includes cognitive impairment and cerebral palsy [1, 8]. Neurotoxicity is progressive, with the development of long-term complications [3]. In fact, despite dietary modifications and the use of ammonia scavengers, chronic intoxication due to the effect of organic acids (methylmalonic and propionic acids), alongside energy deprivation, plays an important role in neurological damage and cognitive outcomes [3, 9]. Of note, the use of sodium phenylbutyrate, an ammonia scavenger, could be deleterious because it chelates glutamine which is already deficient in PA and MMA [4]. While it is crucial to avoid acute hyperammonemia, treatment of chronic (even mild) increases in ammonia remains equally important [4].

Cardiac complications, and cardiomyopathy in particular, may increase mortality in patients with PA, with a number of fatal cases reported [10]. Furthermore, an association with long-QT syndrome has been described [11].

**Fig. 2:**
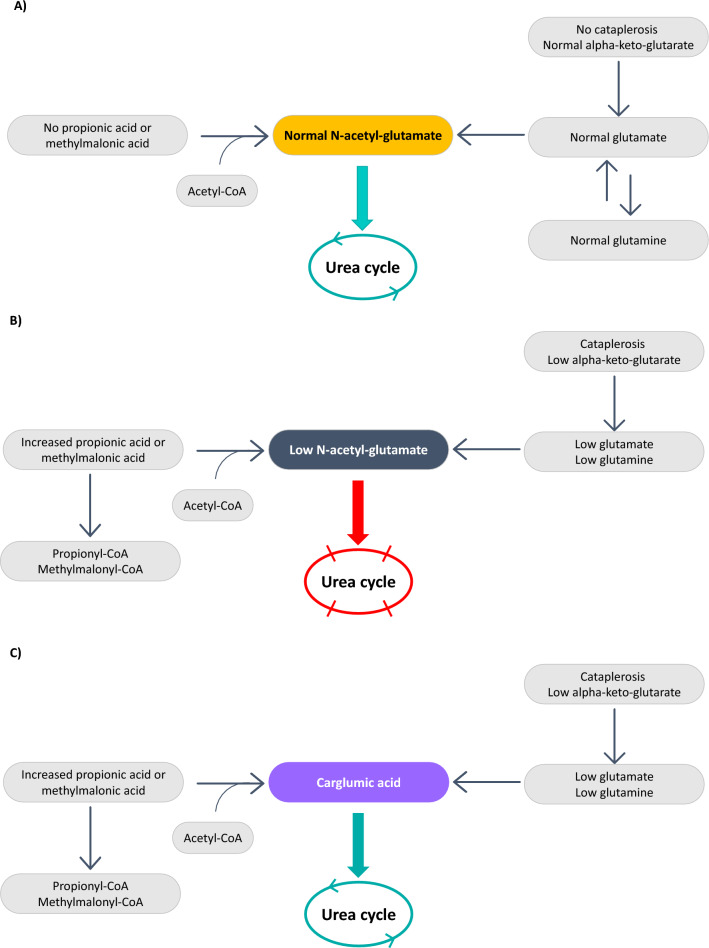
Hyperammonemia in PA and MMA. **A** Normal metabolic function, **B** Pathophysiology of hyperammonemia in PA and MMA, **C** Treatment of hyperammonemia with carglumic acid. CoA, coenzyme A; MMA, methylmalonic aciduria; PA, propionic aciduria

Carglumic acid (N-carbamylglutamate; CGA) is a synthetic structural analog of human NAG, which is the product of the enzyme NAG synthase. NAG is essential for the function of CPS1, therefore, CGA was initially approved for treatment of NAG synthase deficiency [12]. It was subsequently approved in the United States and Europe for treatment of patients with acute hyperammonemia due to PA or MMA (with the limitation of a 7-day course in the United States) [1, 2, 13, 14]. Retrospective studies and case reports have demonstrated that CGA has a good safety profile and effectively restores normal plasma ammonia levels during acute hyperammonemic episodes in patients with organic acidurias (OAs) [2, 15, 16]. In clinical trials, the most common adverse reactions were neutropenia, anemia, vomiting, electrolyte imbalance, decreased appetite, hypoglycemia, lethargy/stupor, encephalopathy and pancreatitis/increased lipase [13]. The dose of CGA should be adjusted in patients with moderate or severe renal impairment [13, 14]. The pathophysiology of hyperammonemia in OAs and the effect of CGA is explained in Fig. 2.

Other treatment options for patients with PA or MMA include conventional therapies for the management of hyperammonemia in urea cycle disorders, with minor appropriate adaptations, which aim to rapidly reduce serum ammonia levels [1, 8]. These include calories (glucose and lipids), dietary protein restriction by oral or enteral nutrition, ammonia scavengers (e.g., sodium benzoate,), dialysis, and liver transplantation (PA or MMA) or combined liver and kidney transplantation (MMA only) [1, 8]. However, their optimal use specifically in patients with PA or MMA currently requires further investigation [1]. In particular, there are concerns regarding sodium phenylbutyrate treatment because it exacerbates the low glutamine levels accompanied by hyperammonemia in OAs [4], therefore its use is not recommended [17]. Currently recommended management of PA and MMA using strict lifelong dietary restrictions is burdensome to patients and their caregivers [3]. The prevention and treatment of OA-associated complications needs to be addressed in order to improve long-term outcomes in these patients [3].

The aim of this expert opinion review was to evaluate clinical data for CGA and to discuss its role, along with other management approaches, in the long-term treatment of PA or MMA.

## Literature search and selection

A literature search of PubMed was conducted to identify publications (i.e., clinical studies, case reports, surveys, and reviews) regarding the use of CGA, transplantation, dietary management, ammonia scavengers, and gene therapies for the chronic treatment of patients with PA or MMA. The authors selected the most relevant studies for discussion in this review, including papers added from the authors’ knowledge.

## Chronic use of carglumic acid

As there is secondary NAG deficiency in PA and MMA, CGA, which acts as NAG substitution therapy, ensures continued elimination of ammonia via the urea cycle by direct allosteric activation of CPS1 [3]. This restoration of urea cycle functioning re-establishes the synthesis of fumaric acid, which could act as an anaplerotic molecule, as it is involved in the Krebs cycle (Fig. 1) [18].

**Fig. 3:**
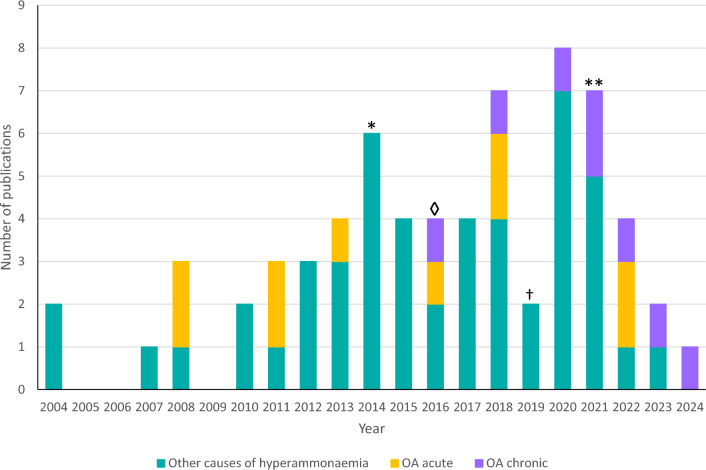
Carglumic acid publications 2004–2024: inherited metabolic disorders. * First guidelines for the diagnosis and management of PA and MMA published. ◊ First study published concerning long-term use of carglumic acid in patients with OAs. † PROTECT Investigators study begins. ** 2021 guidelines for the diagnosis and management of MMA and PA published. MMA, methylmalonic aciduria; OA, organic acidurias; PA, propionic aciduria

While CGA is generally used for treatment of acute hyperammonemia due to PA or MMA, clinical evidence is now accumulating of its efficacy and safety in combination with protein restriction in the chronic setting (Fig. 3). Data regarding the efficacy and safety of long-term CGA maintenance therapy have been published in four clinical studies [19–22], one case series [23], and three case reports [7, 24, 25], with Oxford Centre for Evidence-Based Medicine (OCEBM) levels of evidence of 1–4 [26] (Table 1).

**Table 1:** Details of studies of carglumic acid for the chronic treatment of hyperammonemia due to organic acidurias

Study	OCEBM levels of evidence score (1–5)^a^	Patients	Study design; CGA dose	Pt age at treatment initiation	Efficacy outcomes	Safety outcomes
*Clinical studies*						
Alfadhel (2021) [19]	2	PA or MMA (n = 38)	OL, phase IIIb, RCT (NCT02426775) of std therapy^b^ ± CGA 50 mg/kg/day for 24 mos	≤ 15 yrs	• CGA + std therapy^b^ associated with 51% ↓ in ER admissions over 2 yrs (* p* = 0.0095) and fewer admission days (32.8 vs 51.3 days) • Plasma ammonia levels did not differ with vs without CGA	• No SAEs • AEs included mild vomiting (1 pt receiving CGA + std therapy^b^) and allergy (1 pt receiving std therapy^b^ only)
Kiykim 2021 [21]	3	PA or MMA (n = 21)	Retro study in pts on CGA 12.5–250 mg/kg/day for 3–50 mos	7–146 mos	• 156 hyperammonemia episodes (87 required outpatient dose adjustment, 69 required hospitalization) • Significant ↓ in hospitalizations after treatment initiation (mean 1.207 vs 4.321/year; * p* = 0.013) • Significant ↓ in plasma ammonia levels (mean 55.31 vs 69.64 μmol/L; * p* = 0.021)	• No apparent AEs • No pts discontinued therapy
Burlina 2022 [20]	2	PA or MMA (n = 116–186 [estimated])	Survey conducted in 13 Italian OA referral centers with long-term (> 4 yrs) experience with CGA 50–100 mg/kg/day	0–5 yrs (39% of pts); > 16 yrs (31%)	• 7 centers (54%) used CGA as a chronic treatment in 10–50% of pts and 2 centers (15%) used it in > 50% of pts • After treatment initiation, ↓ in annual decompensation episodes (1–5 episodes in 4 centers; 0 episodes in 9 centers)	• Most centers (70%) reported no difficulties during long-term treatment
Yap 2024 [22]	1	PA or MMA (n = 10)	Prospective, observational study (NCT04176523) of std therapy + CGA for 14–77 mos	2 days–7.88 years	• Median peak ammonia level ↓ from 250 μmol/L to 103 μmol/L • Acute metabolic decompensation events ↓ by median of − 41% • Duration of hospitalization due to metabolic decompensations ↓ in 4/5 evaluable pts	• None reported
*Case series*						
Burlina 2016 [23]	4	PA or MMA with frequent progressive metabolic decompensation episodes and pathologic ammonia levels (n = 8)	CGA 50 mg/kg/day for 7–16 mos	2–20 yrs	• Few decompensation episodes • Ammonia levels significantly ↓ in 5 pts and reduced/maintained in the normal range in 2 pts • Protein intake ↑ by 20–50% in 6 pts • Bodyweight ↑ by 0–6.5 kg	• CGA was well tolerated
*Case reports*						
Tummolo 2018 [25]	4	PA with frequent severe acute decompensation	CGA 100 mg/kg/day for 6 mos then 50 mg/kg/day for up to 6 yrs	9 yrs	• Ammonia levels significantly ↓ (mean 75.7 vs 140.3 µmol/L; * p* < 0.005) • Only 2 acute episodes of decompensation in 6 yrs • Ammonia scavengers gradually discontinued • Protein intake ↑ to 1.2 g/kg/day • BMD improved	• No clinically significant AEs reported
Kido 2020 [7]	4	PA	CGA 100 then 50 mg/kg/day for 26 mos	Neonate	• No hyperammonemia or hospitalization after treatment initiation	None reported
Tubili 2023 [24]	4	MMA with frequent decompensation	CGA 45 then 20 mg/kg/day for > 3 yrs	4 yrs	• Plasma ammonia levels maintained within normal range • Protein intake ↑ to 0.87 g/kg/day • Weight improved (third percentile) and height ↑	None reported

AEs, adverse events; BMD, bone mineral density; CGA, carglumic acid; ER, emergency room; MMA, methylmalonic aciduria; mo(s), month(s); OA, organic aciduria; OCEBM, Oxford Centre for Evidence-Based Medicine; OL, open-label; PA, propionic aciduria; pt(s), patient(s); RCT, randomized, controlled trial; retro, retrospective; SAEs, serious adverse events; std, standard; yr(s), year(s)

^a^1 = highest level of evidence (such as systematic reviews of randomized trials or n-of-1 trials) and 5 = lowest level of evidence (mechanism-based reasoning) [26]

^b^Std therapy = L-carnitine, metronidazole, protein-restricted diet

In an open-label, Phase IIIb, randomized study (NCT02426775) in 38 pediatric patients (mean age approximately 3 years) with PA or MMA, addition of CGA 50 mg/kg/day to standard treatment of L-carnitine, metronidazole, and a protein-restricted diet effectively reduced the long-term frequency of hyperammonemic decompensation episodes (Table 1) [19]. When compared with standard therapy alone, emergency room admissions were significantly reduced with CGA by 51% over 2 years (*p* = 0.0095), and patients receiving CGA reported numerically fewer days of admission than those receiving standard treatment (32.8 vs 51.3 days). CGA was well tolerated, with the only reported adverse event being mild vomiting in one patient, which resolved with slower dose administration [19].

Similarly, a single-center, retrospective study in 21 pediatric patients (mean age 4.4 years) with PA or MMA demonstrated that CGA was effective and well tolerated when administered at a mean long-term dose of 85 mg/kg/day for up to 50 months (Table 1) [21]. In addition to CGA, five patients received sodium benzoate, one received sodium phenylbutyrate, and two received both sodium benzoate and sodium phenylbutyrate. Among 10 evaluable patients, hospitalizations were significantly reduced following CGA initiation compared with before initiation (1.2 vs 4.3 hospitalizations; *p* = 0.013), and, among 11 evaluable patients, plasma ammonia levels were significantly reduced (mean 69.6 vs 55.3 μmol/L; *p* = 0.021) [21]. No apparent adverse effects were reported, and no patients discontinued CGA [21].

The third clinical study was a qualitative survey conducted in 13 Italian metabolic OA referral centers with long-term (> 4 years) experience in the use of CGA 50–100 mg/kg/day in up to an estimated 186 patients with PA or MMA (Table 1) [20]. Results of the survey revealed that patients in 77% of the centers underwent protein restriction. Thirty-nine percent of patients treated with long-term CGA 50–100 mg/kg/day were ≤ 5 years old at treatment initiation. Long-term CGA therapy was associated with reduced frequency of annual hyperammonemic decompensation episodes, with no reported difficulties. An improvement in protein intake due to the reduction in hyperammonemic decompensation episodes was reported by all participating centers [20].

Additionally, the PROTECT study (NCT04176523) is an ongoing, prospective, longitudinal, observational study that is evaluating the clinical outcomes and healthcare utilization of CGA maintenance therapy for the long-term management of PA and MMA (Table 1) [22]. Conducted in Europe, the study is currently enrolling patients with PA or MMA who have been receiving CGA for > 6 months, with follow-up continued for up to 54 months. An interim analysis of PROTECT reported data for ten of 42 enrolled patients with PA (n = 6) or MMA (n = 4) who received CGA for a mean of 36 months (mean age at treatment initiation 28.1 months). All ten patients received dietary restriction prior to initiating CGA. In this analysis, the median peak ammonia level was reduced from 250 μmol/L before treatment to 103 μmol/L after treatment. The frequency of acute metabolic decompensation events with hyperammonemia was reduced by a median of 41% after treatment. CGA treatment also reduced the duration of hospitalization due to metabolic decompensations with hyperammonemia in four of five evaluable patients.

Limitations of the studies above, and the case series and reports, include the relatively small sample sizes, and the lack of formal statistical analysis and study controls. In addition, the CGA dose usually varied within studies, reasons for emergency room visits were often not described, and decompensation events were not defined in any studies except PROTECT [22].

## Outcomes after liver and/or kidney transplantation

Representing a type of partial enzyme replacement [3, 27, 28], transplantation has become established as a treatment option in the management of PA and MMA with increasing clinical experience [3]. Guidelines suggest consideration of liver transplantation in patients with PA or MMA, and combined liver and kidney transplantation in those with MMA to improve metabolic stability [17].

There is evidence that early liver transplantation reduces the occurrence of metabolic decompensations in patients with PA or MMA and the risk of long-term complications and improves patient quality of life [3, 27, 28]. In some patients with MMA, neurologic deterioration may be stabilized and developmental delay may be stabilized or reversed [3]. However, established organ damage cannot be resolved [27]. In a Japanese nationwide study, liver transplantation improved the prognosis and development of patients with MMA after living donor liver transplant, but did not improve kidney and neurological damage [29]. Systematic reviews and meta-analyses of available data have indicated that liver transplantation may improve survival outcomes and reduce disease-related complications in patients with PA or MMA [30, 31].

Kidney transplantation can be used in patients with MMA who have end-stage renal disease caused by tubulointerstitial nephritis [32] and is often combined with liver transplantation [33]. Studies of kidney transplantation are mostly limited to small retrospective studies or case series in patients with MMA who were also undergoing liver transplantation [33–36]. It is uncertain whether kidney transplantation is inferior to combined liver/kidney transplantation for patients with MMA [3].

There are some limitations of using transplantation for the management of PA and MMA. Firstly, there is a lack of prospective and long-term clinical data [3]. Transplantation is supportive only, and is not curative; organ damage cannot be reversed [27, 37, 38]. The burden of a protein-restricted diet, while slightly reduced, is not completely removed with transplantation—dietary restriction may still be required for metabolic stability.[3, 39]. While early transplantation appears to be associated with improved outcomes, the optimal balance of age and other factors is unknown [3]. Improvement of metabolic functioning is limited to the liver; transplantation does not improve other organs or tissues [3, 38]. Furthermore, transplantation does not prevent some long-term complications, including neurologic damage, metabolic decompensation during illness, and cardiomyopathy [3, 34, 40]. Liver transplantation is not able to modify levels of methylmalonic acid and methylcitrate in the cerebrospinal fluid of patients with MMA or PA, which is in accordance with the fact that liver transplant is not a cure [41, 42]. Nevertheless, liver transplantation has a beneficial impact on quality of life [27, 43] and can reduce disease severity [3, 17], even if not curative.

Transplantation may be associated with mortality risk and severe complications [3, 27, 28]. Mortality rates after transplantation are 14–22% for patients with PA and 3–13% for patients with MMA [27, 28, 39], and are particularly high within 14 days after transplantation [28]. Rates of complications associated with transplantation are 54% for patients with PA and 49% for patients with MMA [39]. Graft failure or rejection occurs in 18.9% of PA patients and 10.6% of MMA patients [28], while neurologic complications occur in 2.7% and 9.4% of patients, respectively [28].

Transplantation is also associated with the additional burden of long-term immunosuppression [3, 27, 39]. Furthermore, transplantation procedures are not standardized, due to a lack of defined eligibility criteria or uniform protocols [3].

Ultimately, since transplantation is not curative for these patients, it is important to recognize that any improvement in metabolic control needs to take into consideration the risk of transplant-associated complications and the additional burden of prolonged immunosuppressive therapy post-transplantation [39]. CGA is useful during metabolic crisis and, in the authors’ personal experience, may stabilize the patient prior to liver transplantation.

## Use of dietary (protein) restrictions and/or formula

A protein-restricted diet to reduce the accumulation of toxic metabolites is generally the cornerstone of long-term management of PA and MMA [3, 44–46]. The main dietary goal in such patients should be to prevent toxicity and catabolism, allowing for normal growth and development [6]. Dietary management involves limiting the intake of natural protein to ensure metabolic stability, with supplementation of precursor-free amino acids (valine, isoleucine, methionine, and threonine) to meet nutritional protein requirements [4, 44, 45]. However, the aim is to allow intake of as much natural protein as is tolerated and use of as little amino acid-deficient supplementation to meet age-related recommended dietary allowances. Individual titration of protein tolerance is necessary, based on patient age, growth, metabolic stability, and disorder severity: the diet should be tailor-made and quickly adapted to concurrent illness [4, 44, 45]. Over-restriction or over-treatment should also be avoided during interventions involving dietary restriction. Recommendations regarding the safe levels of protein intake are summarized in Table 2. Studies have shown that early and aggressive nutrition management can help to improve survival and clinical outcomes but cannot exclude decompensation episodes [44].

**Table 2:** Recommended safe levels of protein intake in patients with PA or MMA [3, 4, 44, 46]

	Protein intake g/kg/day		
Age	WHO/FAO/UNU PA/MMA nutrition management guideline [3, 4, 46]	SERN/GMDI PA nutrition management guideline [44]	
	Total protein^a^	Total protein	Natural protein
*Infants*			
1 mo	1.77	1.52–1.82	0.91–1.52
2 mos	1.50	1.52–1.82	0.91–1.52
3 mos	1.36	1.52–1.82	0.91–1.52
4 mos	1.24	1.52–1.82	0.91–1.52
6 mos	1.14–1.31	1.52–1.82	0.91–1.52
7–12 mos	1.14	1.20–1.44	0.72–1.2
*Children/adolescents*			
1–3 yrs	0.90–1.03	1.05–1.26	0.63–1.05
4–6 yrs	0.85–0.89	0.95–1.14	0.57–0.95
7–10 yrs	0.91–0.92	0.95–1.14	0.57–0.95
11–13 yrs	0.92–1.14	0.95–1.14	0.57–0.95
14–16 yrs		0.85–1.02	0.51–0.85
17–18 yrs	0.84–0.87	0.85–1.02	0.51–0.85
*Adults*			
≥ 19 yrs	0.84–0.87	0.80–0.96	0.48–0.80
Pregnancy	Additional intake of 0.7–31.2 g/day	1.10–1.32	0.66–1.10
Lactation	Additional intake of 12.5–20 g/day	1.30–1.56	0.78–1.30

^a^Based on average protein consumption by healthy people; targets should be adjusted for age, sex, mobility, physical activity, and clinical condition

FAO, Food and Agriculture Organization; GMDI, Genetic Metabolic Dietitians International; MMA, methylmalonic aciduria; mo(s), month(s); PA, propionic aciduria; SERN, Southeast Regional Genetics Network; UNU, United Nations University; WHO, World Health Organization; yr(s), year(s)

Despite this, there are several limitations associated with the use of dietary management and protein restriction. Firstly, there is no clear consensus on dietary management and protein restriction and no clear evidence for optimization. Achieving a balance between protein intake sufficient for developmental needs and avoiding toxicity is challenging [3]. Adequate nutrition is essential at all stages of brain development; thus, a lack of some amino acids (mainly isoleucine and valine) and of some micronutrients, particularly vitamin B12, during key phases of development can have permanent effects on the brain [3]. It is important to monitor growth and avoid malnutrition. Commonly used protein formulas have been demonstrated to be of low biologic value [47]. Protein restriction may be associated with a risk of osteoporosis or osteopenia, yet both have only been documented in some patients and osteoporosis seems to be underdiagnosed [11]. Guideline-recommended safe levels of protein intake are based on the needs of healthy people, so may possibly be incorrect for patients with OAs [48, 49]. There is a lack of strong evidence for the efficacy of dietary management and protein restriction in patients with MMA [3, 50] and the role of formulas in PA is considered questionable [51].

Secondly, dietary formulas are comprised of a mix of amino acids without the precursors of propionate, i.e., isoleucine, valine, methionine, and threonine [45, 51] that are not controlled or validated by clinical data/evidence. Their use can induce imbalances in plasma amino acids, which may become deficient (mainly isoleucine and valine) and can severely impair growth and development [3, 52]. Large differences in prescribed and consumed protein intake and in the proportions of natural and synthetic proteins in the diet have been reported [47, 48, 53].

Lastly, a protein-restricted diet can be burdensome for patients, which may affect patient adherence [54]. Adolescents in particular may wish to avoid being perceived as different by their peers [54]. Sometimes nighttime enteral feeding is necessary to maintain calories. Adherence may be better in patients with severe PA or MMA than in those with milder disorders.

## Use of ammonia scavengers

Ammonia scavengers (i.e., sodium benzoate and sodium or glycerol phenylbutyrate) conjugate amino acids (glycine and glutamine respectively), enabling nitrogen excretion while bypassing the urea cycle [4, 6]. Glycine is increased in PA and MMA, thus excretion of the product of conjugation of sodium benzoate to glycine (hippurate), is particularly beneficial [4]. Ammonia scavengers are generally used during decompensation to reduce plasma ammonia levels [6, 16]. As discussed earlier, sodium phenylbutyrate should not be used in this patient population because reduced glutamine levels in OAs may worsen the cataplerotic state [4].

The limitations of this treatment include that the use of ammonia scavengers is off-label in PA and MMA. In particular, there is no clear evidence of benefit with ammonia scavengers in the chronic setting [4]. While considered safe for the treatment of acute hyperammonemia, caution is advised when using ammonia scavengers (sodium phenylbutyrate) in PA and MMA because of potential toxicities due to blocking of the urea cycle through sequestration of coenzyme A, or further depletion of the glutamine/glutamate pool [4, 9, 16].

## Use of gene therapy

Given the genetic origins of PA and MMA (i.e., caused by mutations in *PCCA* or *PCCB* and *MMUT* genes, respectively), treatment of these disorders using gene therapy may be considered logical [55, 56].

Preclinical gene therapy studies have investigated the use of viral gene delivery (adenovirus, lentivirus, or adeno-associated virus), messenger RNA (mRNA), and genome editing gene therapy modalities for the treatment of PA and MMA in mouse models [55–57]. Beneficial outcomes observed in these studies include improved growth, increased survival, and relevant increases or decreases in urea cycle biomarkers [56]. While preclinical results to date appear promising, the use of gene therapies for the treatment of PA or MMA is still in the early stages. Thus far, there are no neurological outcomes data for gene therapy in preclinical models of PA and MMA.

An ongoing phase I/II clinical study is assessing the safety and efficacy of an mRNA-based gene therapy (mRNA-3927) in patients with PA [58]. Sixteen patients have been enrolled so far. Interim results indicate there was a 70% reduction in the risk of metabolic decompensation events among eight patients who reported these events in the 12 months before gene therapy treatment. Another phase I/II clinical study (NCT04899310) is currently assessing the safety, tolerability, pharmacodynamics, and pharmacokinetics of an mRNA-based gene therapy (mRNA-3705) in patients with MMA, with results expected in 2026. The phase I/II SUNRISE study (NCT04581785) evaluating hLB-001, a liver-targeted, recombinant adeno-associated virus vector-based therapy, in patients with MMA, was recently terminated due to a low likelihood of clinical benefit in treated participants.

## Experts’ opinion on the role of carglumic acid

Current guidelines and available recommendations fail to consider potential benefits of long-term CGA on clinical outcomes patients with PA or MMA. The place of CGA in the long-term management of PA and MMA is becoming clearer as more data are published. The addition of CGA, alongside conventional dietary treatment, is safe and effective in the chronic treatment of PA and MMA and may help to overcome some of the issues associated with other therapies used to treat these disorders. It is useful in improving metabolic stability and also as a ‘bridging’ therapy while awaiting liver transplantation. In particular, the addition of CGA to dietary modification is associated with a reduction in hyperammonemic decompensation episodes and admission to hospital, compared with dietary modification alone, in patients at any age. Importantly, an increased protein intake with CGA has been reported in four of the studies discussed in this review [20, 23–25]. Table 3 provides an overview of the benefits and limitations of currently used long-term therapies, highlighting the unique role of CGA.

**Table 3:** Benefits and limitations of therapies for the chronic treatment of hyperammonemia due to organic acidurias

Therapy	Benefits	Limitations
Carglumic acid	• Reduces long-term metabolic decompensation episodes • Reduces emergency room visits • Allows for optimization of protein intake • Well tolerated	• Further data required to fully evaluate benefits and limitations
Transplantation	• Reduces metabolic decompensation episodes • Reduces emergency room visits • Reduces risk of long-term complications • Improves quality of life	• Lack of prospective and long-term data • Not curative • Dietary restrictions still required • Metabolic function improvement limited to liver • Cannot reverse organ damage • Long-term immunosuppression • Complications risk • Mortality risk
Dietary restrictions	• Reduces metabolic decompensation episodes	• No consensus on management • Burdensome for patients • Malnutrition risk • Not suitable long-term
Ammonia scavengers	• Reduce ammonia during metabolic decompensation episodes	• Off-label in PA and MMA • May block urea cycle • May deplete glutamine • Poor palatability
Gene therapy	• May directly address underlying enzymatic defect	• Data too premature to evaluate limitations

MMA, methylmalonic aciduria; PA, propionic aciduria

## Conclusions

CGA has potential as an additional therapy for the chronic management of patients with PA and MMA and should be considered for inclusion in guidelines for the chronic treatment of these disorders.

## Data Availability

Data sharing is not applicable to this article as no datasets were generated or analyzed during the current study.

## Abbreviations

CGA: Carglumic acid
CPS1: Carbamoyl phosphate synthetase-1
MMA: Methylmalonic aciduria
mRNA: Messenger RNA
NAG: N-acetylglutamate
OAs: Organic acidurias
PA: Propionic aciduria
